# Qingchang Wenzhong Decoction Attenuates DSS-Induced Colitis in Rats by Reducing Inflammation and Improving Intestinal Barrier Function via Upregulating the MSP/RON Signalling Pathway

**DOI:** 10.1155/2017/4846876

**Published:** 2017-10-12

**Authors:** Tangyou Mao, Junxiang Li, Lijuan Liu, Weihan Zhao, Yuyue Liu, Kangli Gao, Yi Guo, Tianhong Xie, Ningfei Li, Rui Shi

**Affiliations:** ^1^Beijing University of Chinese Medicine, No. 11, North Third Ring East Road, Beijing 100029, China; ^2^Gastroenterology Department, Dongfang Hospital, Beijing University of Chinese Medicine, No. 6, 1st Section, Fangxingyuan, Fangzhuang, Beijing 100078, China; ^3^China-Japan Friendship Hospital, No. 2, Cherry East Road, Beijing 100029, China; ^4^Gastroenterology Department, The First Affiliated Hospital, Shanxi University of Chinese Medicine, No. 2, Weiyang West Road, Xianyang 712000, China; ^5^Gastroenterology Department, The First Affiliated Hospital, Anhui University of Chinese Medicine, No. 117, Mei Shan Road, Hefei 230031, China

## Abstract

Ulcerative colitis (UC) is a chronic, nonspecific, inflammatory disease for which an effective treatment is lacking. Our previous study found that Qingchang Wenzhong Decoction (QCWZD) can significantly improve the clinical symptoms of UC and ameliorate dextran sulphate sodium- (DSS-) induced ulcerative colitis in rats by downregulating the IP10/CXCR3 axis–mediated inflammatory response. The purpose of the present study was to further explore the mechanism of QCWZD for UC in rats models, which were established by 7-day administration of 4.5% dextran sulphate sodium solution. QCWZD was administered daily for 7 days; then we determined the serum macrophage-stimulating protein concentration (MSP) and recepteur d'origine nantais (RON) expression and its downstream proteins (protein kinase B [Akt], phosphorylated [p] Akt, occludin, zona occluden- [ZO-] 1, and claudin-2) in colon tissue using Western blotting and quantitative polymerase chain reaction. In DSS-induced UC, QCWZD significantly alleviated colitis-associated inflammation, upregulated serum MSP expression and RON expression in the colon, reduced the pAkt levels, promoted colonic occluding and ZO-1 expression, and depressed claudin-2 expression. In conclusion, the MSP/RON signalling pathway plays an important role in the pathogenesis of UC by involving the inflammatory response and improving intestinal barrier function. QCWZD appears to attenuate DSS-induced UC in rats by upregulating the MSP/RON signalling pathway.

## 1. Introduction

Ulcerative colitis (UC) is a nonspecific, chronic inflammation of the colon and rectum, primarily of the mucosal and submucosal layers [1, 2]. UC frequently occurs in young people and is characterized by abdominal pain, diarrhoea, and bloody mucopurulent stool [3]. UC-related complications including toxic giant colon, bleeding, perforation, and cancer seriously affect quality of life [4]. Thus, the World Health Organization lists UC as a miscellaneous problem [5].

The number of patients with UC is increasing annually in Asia, and the incidence of UC has increased more than 3 times in 10 years in China [6]. Because of delayed healing, the rate of UC recurrence is very high. In addition, the detection rate of UC-associated colorectal cancer (UC-CRC), which is one of the most serious complications, has increased, and UC-CRC now accounts for 10–15% of deaths in patients with UC [7]. The disease process that leads to UC-CRC involves “continuous intestinal inflammation, suspicious atypical hyperplasia, a low degree of atypical hyperplasia, and highly atypical hyperplasia and cancer”; however, the later steps can be skipped [8]. Moreover, the progression through “inflammation to atypical hyperplasia to cancer” in patients with UC is more rapid than the progression of “adenoma to adenocarcinoma” in the general population [9, 10]. Thus, as an independent risk factor, intestinal inflammation is the first step in UC-CRC, and the risk of CRC increases with the severity of inflammation in patients with atypical hyperplasia or chronic UC. Furthermore, the length of the disease course is a key factor for cancer in patients with UC; with disease lasting 10 years and more, the average incidence rate of cancer increases exponentially [11]. Therefore, controlling inflammation in UC not only improves quality of life and work efficiency, but also prevents the development and reduces the incidence of UC-CRC.

The macrophage-stimulating protein (MSP)/recepteur d'origine nantais (RON) signalling pathway plays a critical role in the inflammatory process [12–14]. MSP is a hepatocyte-like cell growth factor and belongs to the plasminogen related growth factor family. Recent evidence indicates that MPS is deeply involved as an inhibitory factor in the endogenous inflammatory response. MSP is secreted by the hepatic cells into the blood as an inactive single strand (pro-MSP). In various pathological conditions, serum- and membrane-bound proteases can activate pro-MSP to become biologically active mature MSP, which is composed of alpha chains (60 kDa) and beta chains (30 kDa) at the RON binding site.

As the only specific binding protein of MSP, RON is activated when bound by MSP. Then, it not only exerts anti-inflammatory effects by inhibiting production of proinflammatory cytokines from peritoneal macrophages [15], but also maintains the homeostasis of the intestinal epithelium; regulates the proliferation, survival, and migration of intestinal epithelial cells; and promotes the repair of injured intestinal epithelial cells and mucosal barrier by regulating multiple downstream signalling cascades [16, 17]. Therefore, decreased MSP/RON pathway function, which decreases the anti-inflammatory processes and repair of intestinal mucosa, is one of the mechanisms of UC. The agents that activate the MSP/RON pathway could be drug targets for UC treatment.

Although research for UC has made great progress in recent years, effective treatment is still lacking. Aminosalicylates and glucocorticosteroids are currently the first-line drugs for mild-moderate and moderate-severe UC [18], respectively. The issues with these drugs include lack of tolerance to the drug and side effects such as the required treatment length and high recurrence rates. In addition, the efficacy and safety of immunomodulators and antibiotics, which are currently in the clinical trial stages, require further evaluation [19]. Thus, there is an increasing need for the development of more effective and less toxic agents for the treatment of UC.

We have already shown that QCWZD not only significantly improves the symptoms of active UC in patients with diarrhoea with mucous, pus, and blood [20], but also reduces damage to mucosal epithelial cells in rats models by downregulating the IP10/CXCR3 axis–mediated inflammatory response [21] and inhibiting proinflammatory cytokines (e.g., interleukin-6/tumour necrosis factor-alpha) secreted by macrophages [22]. Contrary to the destructive mechanism of IP10/CXCR3 axis, the MSP/RON signalling pathway plays a protective role in the pathogenesis of ulcerative colitis. So from the opposite perspective, we explored the possible protective mechanism of QCWZD in rats with DSS-induced UC, with the aim of providing a comprehensive experimental basis for the use of QCWZD as a potential therapeutic agent in the treatment of UC.

## 2. Materials and Methods

### 2.1. Qingchang Wenzhong Decoction Preparation

QCWZD formula granules, purchased from the Dong Fang Hospital pharmacy of the Beijing University of Chinese Medicine, were composed of eight commonly used herbs: 6 g Huanglian (coptis), 10 g Pao Jiang (ginger), 15 g Kushen (matrine), 6 g Qingdai (indigo), 30 g Diyutan (sanguisorba carbon), 6 g Mu Xiang (wood), 6 g Sanqi (pseudo-ginseng), and 6 g Gan Cao (licorice). Mesalazine was purchased from Losan Pharma GmbH, Germany.

### 2.2. Animals and Experimental Procedure

Sixty male Sprague-Dawley rats (weight, 180–220 g) were purchased from the Experimental Animal Science and Technology Co. Ltd. (Beijing, China; certificate no. SCXK-2011-0004) and raised in a specific pathogen-free animal room at the Research Institute of Chinese Medicine in the Chinese Academy of Traditional Chinese Medicine (Beijing, China), which is a temperature- (20–24°C), humidity- (50–60%), and light-controlled environment (12-h light/dark cycle), with ad libitum access to rodent feed and water. We used DSS solution (4.5%), which would simulate human pathogenesis and symptoms, to develop the rat colitis model.

After adaptive feeding for 1 week, the rats were randomly divided into five groups of 10 rats each for the 7-day intervention: blank control group, treated with 2 mL distilled water and free access to tap water; DSS-treated group, treated with 2 mL distilled water and free access to 4.5% DSS; low-dose QCWZD group, treated with 2 mL of 0.3 g/kg body weight (bw) QCWZD and free access to 4.5% DSS; medium-dose QCWZD group, treated with 2 mL of 0.6 g/kg bw QCWZD and free access to 4.5% DSS; high-dose QCWZD group, treated with 2 mL of 1.2 g/kg bw QCWZD; mesalazine group, treated with 2 mL of 0.03 g/kg bw mesalazine and free access to 4.5% DSS. We observed the general condition, weight, and faecal occult blood on a daily basis.

### 2.3. Reagents

DSS (MW36–50 KDa; MP Biomedical, Burlingame, CA, USA), an ultraviolet spectrophotometer (NANODROP 2000; Thermo Scientific, Wilmington, DE, USA), and enzyme-linked immunosorbent assay (ELISA) kits for MSP were purchased from Shanghai BlueGene Biotechnology Co., Ltd. (Shanghai, China) in addition to Anti-RON (ab125283), anti-pAkt (S473) (ab18206), anti-Akt (ab5893), anti-occludin (Ab31721), anti-ZO-1 (SC-8146), and anti-claudin-2 (ab12593).

### 2.4. Detection of Serum Macrophage-Stimulating Protein Levels

The possible protective mechanism of QCWZD on UC was evaluated by measuring the MSP using ELISA kits, according to the manufacturer's instructions (Multiskan MK3; Thermo Scientific, Rockford, IL, USA).

### 2.5. Analyses of RON, Akt, p-Akt, Occludin, ZO-1, and Claudin-2 Expression

To investigate if QCWZD has a regulatory effect on RON expression, we conducted Western blot analysis, followed by quantitative polymerase chain reaction (qPCR). Then, to further clarify RON activity levels, we detected the levels of Akt and phosphorylated (p) Akt in the PI3K/Akt signalling pathway. Meanwhile, dysfunction of the MSP/RON pathway leads to disruption of the intestinal mucosal barrier; we determined the levels of occludin, zona occluden- [ZO-] 1, and claudin-2.

Protein was isolated from ice-cold colon tissues as described previously [23]; then, the protein was separated using 10% sodium dodecyl sulphate-polyacrylamide gel electrophoresis and transferred to polyvinylidene difluoride membranes. Membranes were immunoblotted with primary antibodies that recognized RON (1 : 1000), Akt (1 : 1000), p-Akt (1 : 500), occludin (1 : 1000), ZO-1 (1 : 500), claudin-2 (1 : 500), and GAPDH (1 : 1000) antibodies (TDY Biotech, Beijing, China). Peroxidase-conjugated secondary antibodies [goat polyclonal secondary antibody to rabbit (111-035-003), Jackson, USA] were also used. Densitometry was used to quantitate protein band intensities using the Gel Image System ver. 4.00 (Tanon, China).

Total RNA was extracted from the colon tissue samples after the 7 days of therapy, and the purity and concentration of the PCR were calculated. After reverse transcription, PCR amplification was performed using the TRIzol® method (TRIzol reagent; Invitrogen Life Technologies, Carlsbad, CA, USA). The primer sequences were 5′- TGCTTATTCCCTCTCCCCGA -3′/5′- CCTCGGCTAGGAGCATCTTG -3′ for RON, 5′- CAGACACCTTTGCACTTGGC -3′/5′- CTTGAGTAGGACCCCGAGGA -3′ for occludin, 5′- ATGACCGAGTCGCAATGGTT -3′/5′- TCTATCCCTTGCCCAGCTCT -3′ for ZO-1, 5′- GTCAGCTTGCCAGAGACACT -3′/5′- TTCGCTTGTCTTTTGGCTGC -3′ for claudin-2, and 5′- CCCATCTATGAGGGTTACGC -3′/5′- TTTAATGTCACGCACGATTTC -3′ for GAPDH (CWbio, Beijing, China). Changes in the target genes were determined using the 2−ΔΔCt method [24].

### 2.6. Statistical Analysis

SPSS v18.0 (IBM Corp, Armonk, NY, USA) was used for statistical analyses. Data are expressed as mean ± standard error. The data were compared between groups using one-way analysis of variance (ANOVA), followed by Student's *t*-tests. *P* < 0.05 was considered statistically significant.

## 3. Results

### 3.1. Effects of QCWZD on Serum MSP Level

**Figure 1:**
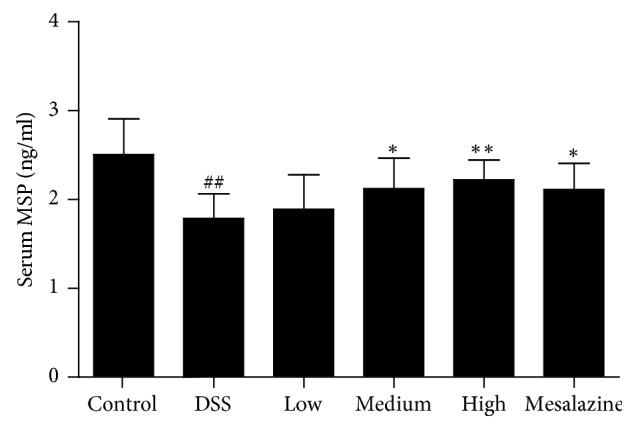
Effects of QCWZD on serum MSP level. Control, blank control group; DSS, DSS-treated group; low, low-dose QCWZD group; medium, medium-dose QCWZD group; high, high-dose QCWZD group; mesalazine, mesalazine group. ^##^*P* < 0.01 versus the control group; ^*∗∗*^*P* < 0.01, ^*∗*^*P* < 0.05 versus the DSS group (*n* = 10 per group).

All animals tolerated the entire experiment and no deaths occurred. After intrarectal administration of QCWZD and mesalazine for 7 days, all rats were killed. Then we determined serum MSP level. As shown in Figure 1, serum MSP levels were significantly lower in the DSS group than in the control group (1.789 ± 0.2744 ng/ml in the DSS group versus 2.506 ± 0.4025 ng/ml, *P* < 0.01). Compared with the DSS group, the medium-dose QCWZD group (2.121 ± 0.3428 ng/ml), the high-dose QCWZD group (2.223 ± 0.2219 ng/ml), and the mesalazine group (2.111 ± 0.2958 ng/ml) showed significant activation (*P* < 0.05, *P* < 0.01, resp.) (Figure 1).

### 3.2. QCWZD Regulated Colonic RON and RON mRNA Expression in DSS-Induced UC Rats

**Figure 2:**
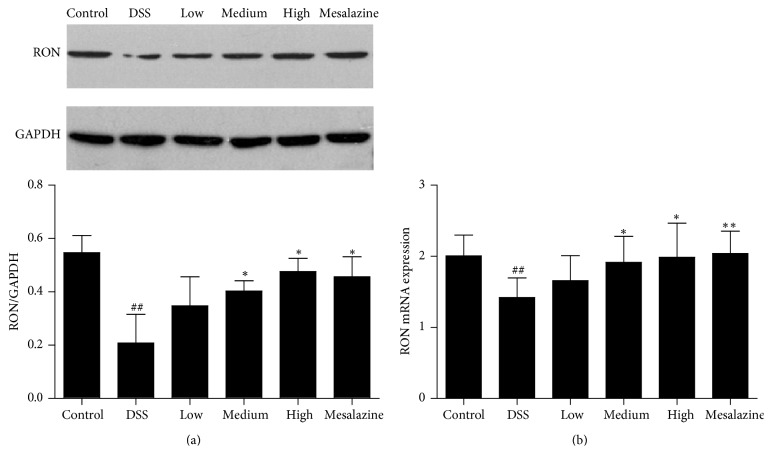
QCWZD regulated colonic RON (a) and RON mRNA (b) expression in DSS-induced UC rats. Control, blank control group; DSS, DSS-treated group; low, low-dose QCWZD group; medium, medium-dose QCWZD group; high, high-dose QCWZD group; mesalazine, mesalazine group. ^##^*P* < 0.01 versus the control group; ^*∗∗*^*P* < 0.01, ^*∗*^*P* < 0.05 versus the DSS group (*n* = 10 per group).

As the only specific binding protein of MSP, RON is activated when bound by MSP; then we analysed RON level. Western blot analyses showed that the RON level was significantly lower in the DSS group than in the control group (*P* < 0.01, Figure 2(a)). Significantly higher RON levels were present in medium-dose QCWZD, high-dose QCWZD, and mesalazine groups than in the DSS group (*P* < 0.05, Figure 2(a)).

Next, we measured RON gene expression to confirm the effects of QCWZD on the colon. The qPCR analyses showed significantly lower RON mRNA levels in the DSS group than in the control group (*P* < 0.01, Figure 2(b)), and the medium-dose QCWZD, high-dose QCWZD, and mesalazine groups increased the RON gene expression compared to that in the control group (*P* < 0.05, *P* < 0.01, resp., Figure 2(b)).

### 3.3. QCWZD Regulated Colonic PI3K/Akt Signalling Pathway in DSS-Induced UC Rats

**Figure 3:**
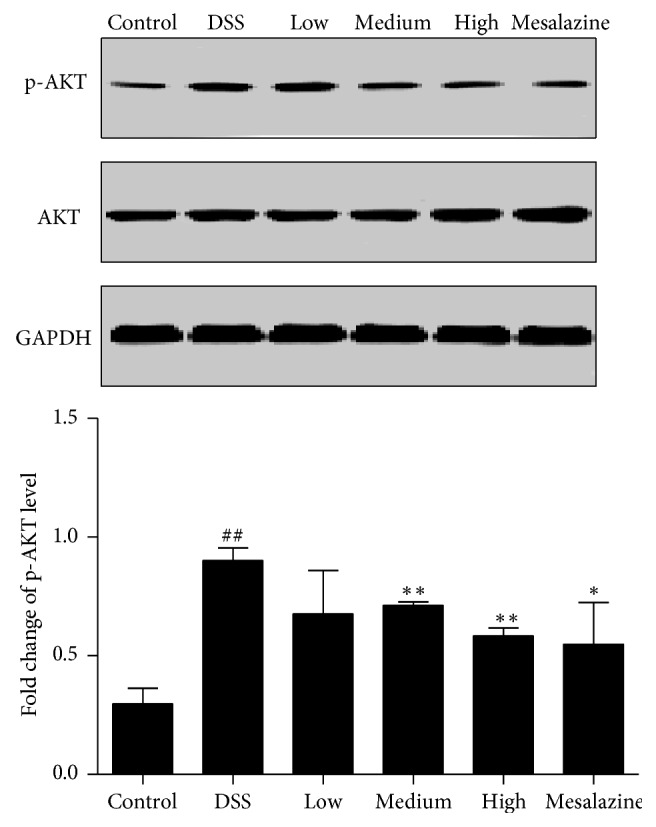
QCWZD regulated colonic PI3K/Akt signalling pathway in DSS-induced UC rats. Control, blank control group; DSS, DSS-treated group; low, low-dose QCWZD group; medium, medium-dose QCWZD group; high, high-dose QCWZD group; mesalazine, mesalazine group. ^##^*P* < 0.01 versus the control group; ^*∗∗*^*P* < 0.01, ^*∗*^*P* < 0.05 versus the DSS group (*n* = 10 per group).

PI3K/Akt signalling pathway is an important regulatory pathway of RON activity; therefore, the level of PI3K/Akt expression can reflect RON activity [25], so we examined fold change of p-Akt level. As shown in Figure 3, the fold change of p-Akt level was significantly higher in the DSS group than in the control group (*P* < 0.01) and significantly lower in the medium-dose QCWZD, high-dose QCWZD, and mesalazine groups than in the DSS group (*P* < 0.05, *P* < 0.01, resp.).

### 3.4. QCWZD Increased Colonic Occludin and Occludin mRNA Expression in DSS-Induced UC Rats

**Figure 4:**
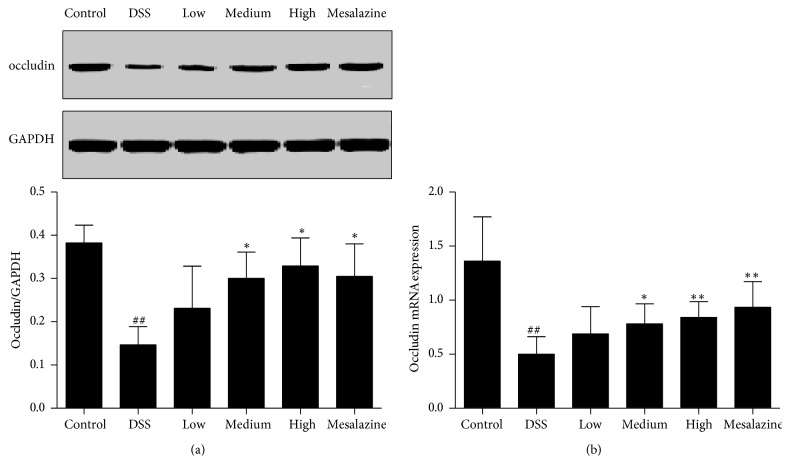
QCWZD increased colonic occludin (a) and occludin mRNA (b) expression in DSS-induced UC rats. Control, blank control group; DSS, DSS-treated group; low, low-dose QCWZD group; medium, medium-dose QCWZD group; high, high-dose QCWZD group; mesalazine, mesalazine group. ^##^*P* < 0.01 versus the control group; ^*∗∗*^*P* < 0.01, ^*∗*^*P* < 0.05 versus the DSS group (*n* = 10 per group).

Disruption of tight junctions is an important basis for the pathogenesis of ulcerative colitis, and dysfunction of the MSP/RON pathway leads to disruption of the intestinal mucosal barrier, so occludin, a major protein in tight junctions, was examined. We found distinctly decreased occludin and occludin mRNA expression in the DSS group compared to that in the control group (*P* < 0.01, Figures 4(a) and 4(b)). Nevertheless, occludin and occludin mRNA expression were significantly induced in DSS-induced UC rats treated with medium-dose QCWZD, high-dose QCWZD, and mesalazine (*P* < 0.05, *P* < 0.01, resp., Figures 4(a) and 4(b)).

### 3.5. QCWZD Upregulated Colonic ZO-1 and ZO-1 mRNA Expression in DSS-Induced UC Rats

**Figure 5:**
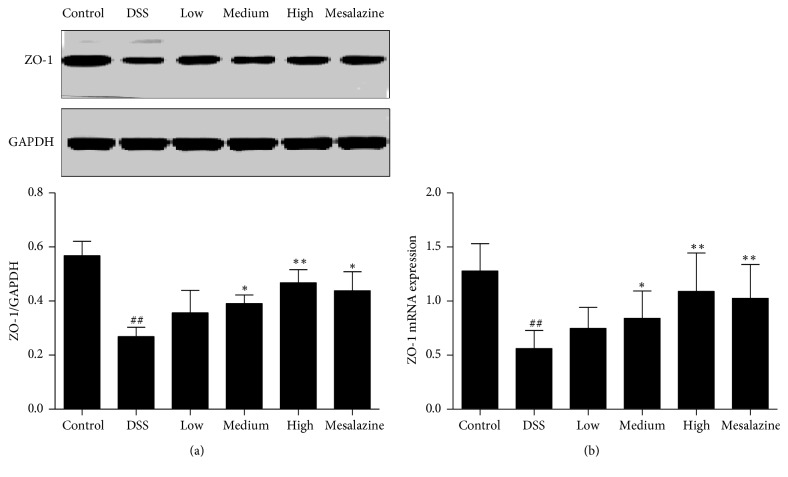
QCWZD upregulated colonic ZO-1 (a) and ZO-1 mRNA (b) expression in DSS-induced UC rats. Control, blank control group; DSS, DSS-treated group; low, low-dose QCWZD group; medium, medium-dose QCWZD group; high, high-dose QCWZD group; mesalazine, mesalazine group. ^##^*P* < 0.01 versus the control group; ^*∗∗*^*P* < 0.01, ^*∗*^*P* < 0.05 versus the DSS group (*n* = 10 per group).

As a peripheral membrane protein in tight junctions, ZO-1 levels were analysed by Western blot. Compared to the control group, the DSS group showed significantly decreased ZO-1 expression (*P* < 0.01, Figure 5(a)), and the medium-dose QCWZD, high-dose QCWZD, and mesalazine showed promotion effects (versus the DSS group, *P* < 0.05, *P* < 0.01, resp., Figure 5(a)). Similarly, we found that the expression of the ZO-1 gene in the DSS group was decreased compared to that in the control group (*P* < 0.01, Figure 5(b)), and compared to the DSS group, medium-dose QCWZD, high-dose QCWZD, and mesalazine distinctly increased ZO-1 gene expression distinctly (*P* < 0.05, *P* < 0.01, resp., Figure 5(b)).

### 3.6. QCWZD Downregulated Colonic Claudin-2 and Claudin-2 mRNA Expression in DSS-Induced UC Rats

**Figure 6:**
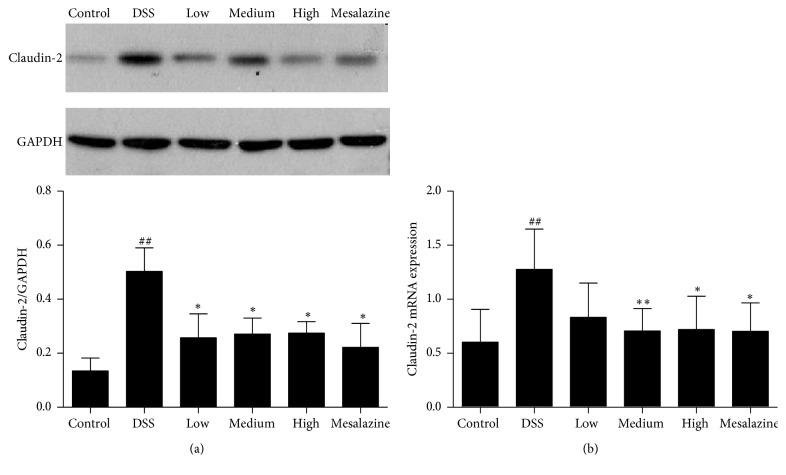
QCWZD upregulated colonic claudin-2 (a) and claudin-2 mRNA (b) expression in DSS-induced UC rats. Control, blank control group; DSS, DSS-treated group; low, low-dose QCWZD group; medium, medium-dose QCWZD group; high, high-dose QCWZD group; mesalazine, mesalazine group. ^##^*P* < 0.01 versus the control group; ^*∗∗*^*P* < 0.01, ^*∗*^*P* < 0.05 versus the DSS group (*n* = 10 per group).

Upregulation of the tight junction protein claudin-2 is considered to contribute to the dysregulation of the epithelial barrier [26], so we analysed claudin-2 expression. Statistical analysis showed that, in rat with DSS-induced colitis, claudin-2 expression in the DSS group was higher than that in the control group (*P* < 0.05, Figure 6(a)) but was significantly lower than that in the QCWZD and mesalazine groups. And then we examined claudin-2 gene expression. We found that the claudin-2 gene expression was significantly higher than that in the control group. Treatment with medium-dose QCWZD, high-dose QCWZD, and mesalazine significantly reduced the claudin-2 gene expression (*P* < 0.05, *P* < 0.01, resp., Figure 6(b)).

## 4. Discussion

Our previous studies have shown that QCWZD had a prominent therapeutic effect on DSS-induced UC in rats, and we have made a preliminary study on the mechanism of QCWZD in treating ulcerative colitis from IP10/CXCR3 axis–mediated inflammatory response. Contrary to the destructive mechanism of IP10/CXCR3 axis, the MSP/RON signalling pathway plays a protective role in ulcerative colitis, so we continued to explore the possible protective mechanism of QCWZD in rats with DSS-induced UC from the opposite perspective, with the aim of providing a comprehensive experimental basis for the use of QCWZD.

Although the aetiology and pathogenesis of UC have not been fully elucidated [27], it is commonly believed that immune inflammatory response and intestinal barrier function destruction play important roles in the pathogenesis of UC [28–30]. The MSP/RON signalling pathway is closely related to cytokine expression [31, 32], and the absence or inhibition of RON can reduce the migration and reproduction of epithelial cells and increase the production and release of inflammatory factors, which are more sensitive to external factors, thereby resulting in more severe colitis. In contrast, the activation of RON, via the binding of MSP to RON, regulates multiple downstream signalling cascades, which could inhibit peritoneal macrophages and exert anti-inflammatory effects, while regulating the proliferation, survival, and migration of and promoting the repair of injury to intestinal epithelial cells. In the present study, serum MSP levels were significantly lower in the DSS group than in the control group; similarly, the expression of RON mRNA and protein, which are MSP-specific receptors, in the colon tissue was significantly lower. In addition, our previous studies found that the DAI and MPO activity of the rats with UC were higher than that in the control group and with the 7-day QCWZD treatment; the DAI and MPO levels were significantly lower, which were negatively correlated with MSP and RON expression (Figures 1 and 2). This interesting phenomenon further supports the fact that the MSP/RON signalling pathway is closely related with the degree of inflammation and plays an important role in the pathogenesis of UC.

The PI3K/Akt signalling pathway is closely associated with the production and release of cytokines, which play an important role in abnormal immune responses [33–35]. Previous experiments have confirmed that the PI3K inhibitor Wortmannin inhibits the activation of PI3K and reduces the activity of Akt in the main downstream target in the colonic mucosa of patients with UC to achieve effective treatment [36]. Based on previous studies, we speculated that activated RON transduces a variety of signals that regulate different downstream pathways, including the PI3K/Akt signalling pathway, and has anti-inflammatory and epithelial repair effects. Thus, PI3K/Akt can be used as an indicator of the MSP/RON signalling pathway activity. In the present study, the fold changes of p-Akt level were significantly higher in the DSS group, indicating enhanced activity of the PI3K/Akt signalling pathway. With the 7-day QCWZD treatment, the activity was significantly lower (Figure 3).

The intestinal mucosal barrier is the first barrier of self-protection of the colon, so barrier dysfunction is an important material basis for the pathogenesis of ulcerative colitis [37, 38]. Recent studies have shown that altered expression of the tight junction protein is considered to contribute to the dysregulation of the epithelial barrier [2, 37, 39], and activation of the MSP/RON pathway differentially regulates tight junction function [40]. Therefore, we evaluated the expression of colonic tight junction protein. In this study, we chose occludin, ZO-1, and claudin-2 as the representative proteins of tight junctions. Occludin is a crucial tight junction protein in the pathogenesis of ulcerative colitis [41], which plays an important role in maintaining the integrity of intestinal mucosal barrier. As a peripheral membrane tight junction protein, ZO-1 can physically bind the distal C-terminus of occluding, so as to ensure the accurate connection of occludin protein [42]. With just the opposite, upregulation of claudin-2 is considered to contribute to the dysregulation of the epithelial barrier [26]. In the present study, compared with the control group, the expression of tight junction associated proteins including occludin and ZO-1 was decreased (Figures 4 and 5); claudin-2 was increased in DSS-induced UC rats (Figure 6), indicating that intestinal barrier function was damaged seriously. Treatment with QCWZD and mesalazine dependently increased occludin and ZO-1 expression (Figures 4 and 5) and decreased claudin-2 expression (Figure 6). These results suggest that QCWZD plays an important role in the protection of intestinal barrier function.

In the present study, we found that MSP/RON signalling pathway plays an important role in the pathogenesis of UC by involving the inflammatory response of macrophages and repairing epithelial cells. Intragastric administration of QCWZD significantly upregulated serum MSP level and RON expression in the colon, reduced the pAkt levels, increased colonic occluding and ZO-1 expression, and downregulated claudin-2 expression, so as to inhibit the intestinal inflammation, improve the intestinal mucosal barrier function, and finally achieve the purpose of repairing intestinal mucosa and treating ulcerative colitis.

## 5. Conclusions

Our results show that QCWZD ameliorates DSS-induced UC in rats mainly by reducing inflammation and improving intestinal barrier function via upregulating the MSP/RON signalling pathway.
